# Cinnamon Extract and Probiotic Supplementation Alleviate Copper-Induced Nephrotoxicity via Modulating Oxidative Stress, Inflammation, and Apoptosis in Broiler Chickens

**DOI:** 10.3390/ani11061609

**Published:** 2021-05-29

**Authors:** Sara T. Elazab, Nahla S. Elshater, Asmaa T. Y. Kishaway, Huda A. EI-Emam

**Affiliations:** 1 Department of Pharmacology, Faculty of Veterinary Medicine, Mansoura University, Mansoura 35516, Egypt; 2 Reference Laboratory for Veterinary Quality Control on Poultry Production, Animal Health Research Institute, Agriculture Research Center, Dokki, Giza 12618, Egypt; nahlaelshater80@gmail.com; 3 Department of Nutrition and Clinical Nutrition, Faculty of Veterinary Medicine, Zagazig University, Al Sharqia Governorate 44519, Egypt; No.dispair2000@gmail.com; 4 Department of Husbandry and Development of Animal Wealth, Faculty of Veterinary Medicine, Mansoura University, Mansoura 35516, Egypt; hudagenetic@yahoo.com

**Keywords:** cinnamon, copper, probiotic, nephrotoxicity, oxidative stress, inflammation

## Abstract

**Simple Summary:**

Copper (Cu), an essential trace element required for many biological processes inside the body, may cause deleterious effects on several body organs when its administration exceeds the tolerable upper intake level. Recently, great attention has been given to the use of natural compounds that are rich sources of biologically active molecules to prevent and treat many diseases. Therefore, this study was designed to explore the possible protective effects of cinnamon extract and probiotic against nephrotoxicity caused by overdose of Cu in broiler chickens. The whole experiment lasted 6 weeks. Cinnamon extract and probiotic showed remarkable antioxidant, anti-inflammatory, and antiapoptotic properties against the toxic effects of excess Cu in renal tissues of chickens. Based on our results, we conclude that cinnamon extract and/or probiotic can serve as an effective therapeutic option to decrease the renal injury caused by Cu poisoning in broiler chickens.

**Abstract:**

The present study aimed to assess the potential protective effects of cinnamon (Cinnamomum zeylanicum, Cin) and probiotic against CuSO_4_-induced nephrotoxicity in broiler chickens. One-day-old Cobb chicks were assigned into seven groups (15 birds/group): control group, fed basal diet; Cin group, fed the basal diet mixed with Cin (200 mg/kg); PR group, receiving PR (1 g/4 L water); Cu group, fed the basal diets mixed with CuSO_4_ (300 mg/kg); Cu + Cin group; Cu + PR group; and Cu + Cin + PR group. All treatments were given daily for 6 weeks. Treatment of Cu-intoxicated chickens with Cin and/or PR reduced (*p* < 0.05) Cu contents in renal tissues and serum levels of urea, creatinine, and uric acid compared to the Cu group. Moreover, Cin and PR treatment decreased lipid peroxidation and increased antioxidant enzyme activities in chickens’ kidney. Additionally, significant reduction (*p* < 0.05) in the mRNA expression of tumor necrosis factor alpha (TNF-α), interleukin (IL-2) and Bax, and in cyclooxygenase (COX-II) enzyme expression, and significant elevation (*p* < 0.05) in mRNA expression of IL-10 and Bcl-2 were observed in kidneys of Cu + Cin, Cu + PR, and Cu + Cin + PR groups compared to Cu group. Conclusively, Cin and/or PR afford considerable renal protection against Cu-induced nephrotoxicity in chickens.

## 1. Introduction

Copper (Cu), an essential micronutrient, exerts a pivotal role in various metabolic processes in the body such as respiration, reactive oxygen species (ROS) quenching, and hematogenesis [1,2]. It acts as a cofactor for a multitude of enzymes (Zinc superoxide dismutase, cytochrome-c oxidase, p-hydroxyphenylpyruvate hydrolase, tyrosinase) required for several biochemical reactions [3,4]. Despite its valuable biological action as an essential trace mineral, Cu can induce devastating toxic effects on multiple body organs when its administration surpasses the tolerable upper intake level [5]. The ubiquitous usage of Cu in agricultural and industrial fields may increase the likelihood of its environmental pollution [6]. What’s more, the long-term exposure to Cu through consuming it in the form of mineral supplements and drinking water may potentially lead to its excessive accumulation in birds and mammals [7]. Former research has revealed that high concentrations of Cu were detected in avian species, which consequently led to deterioration of their physiological processes [8].

The kidney is considered one of the major target organs of Cu poisoning in the animal body due to its circulation and excretion function [9]. The suggested mechanism beyond kidney injury caused by copper toxicity is through inducing oxidative stress. Cu poisoning provokes the generation of free radicals, which in turn leads to a status of oxidant–antioxidant imbalance [9,10]. Therefore, utilizing exogenous antioxidants may alleviate Cu-induced toxicity in kidney and other tissues.

Today, considerable attention has been paid toward the use of natural plant-derived antioxidants as a remedy for different ailments, owing to their safety, availability, and consumer acceptability [11,12]. Furthermore, several studies have revealed that medicinal plants and their constituents can confer a mitigative effect against heavy metal toxicity [13,14]. Cinnamon (Cinnamomum zeylanicum, Cin), a member of Lauraceae family, is a commonly utilized plant in folk medicine with several bioactive effects [15]. Its great content of polyphenolic substances qualified it to serve as a nutritional supplement of natural antioxidants [16,17]. Polyphenolic derivatives are characterized by having ROS sweeping activity and metal chelating effect [18]. In addition to its antioxidant activity, Cin has been recorded to have other pharmacological actions, for instance, anticancer, hypotensive, antimicrobial, anti-inflammatory, insecticidal, and cholesterol-reducing effects [19,20,21,22,23]. Moreover, it has been proven that the entire Cin extract could afford protection against cadmium, gentamicin, diclofenac sodium, oxytetracycline, glutamate, and bisphenol-induced oxidative injury [17,24,25,26,27].

Probiotics (PR), living nonharmful bacterial organisms, are feed additives that promote host health via adjusting gut microbial balance [28,29]. In fact, PR have been recorded to enhance the immune system and increase vitamin synthesis in the body [30]. Over the last few years, previous investigators have revealed that these PR can bind and eliminate heavy metals such as cadmium and lead from the body [31,32,33]. To the best of our knowledge, the effects of Cin and PR on Cu-induced kidney damage have not been studied to date. Hence, this study was conducted to elucidate the potential protective effects of Cin and PR against renal injury induced by Cu toxicity in broiler chickens.

## 2. Materials and Methods

### 2.1. Materials

Copper (II) sulphate pentahydrate (CuSO_4_·5H_2_O; CAS number 7758-99-8) was purchased from Sigma Aldrich Co. (St. Louis, MO, USA). The PR (Vitabiotic^®^) was manufactured by Vital Therapeutics & Formulations Pvt Ltd., Hyderabad, India. Each gram of this PR contains a mixture of the following alive bacterial flora: Lactobacillus acidophilus (2.06 × 108 CFU), Lactobacillus plantarum (1.26 × 108 CFU), Lactobacillus casei (2.06 × 108 CFU), Lactobacillus bulgaricus (2.06 × 108 CFU), Streptococcus thermophillis (4.10 × 108 CFU), and Streptococcus faecium (5.40 × 108 CFU). Nitric acid (HNO_3_) and perchloric acid (HCLO4) were purchased from Merck Co. (Darmstadt, Germany).

### 2.2. Plant Extract Preparation

The extraction of Cin plant was carried out following the method published previously [34]. Briefly, the Cin barks (Cinnamomum zeylanicum) were obtained from an herbal drug store (Mansoura, Egypt) and identified by Prof. Dr. Ibrahim Mashaly (Botany Department, Faculty of Science, Mansoura University, Egypt). The barks were dried and ground into a fine powder using a milling machine. Then, the obtained powder was soaked in 70% methanol at 25 °C for 48 h. The previous step was repeated three times. Afterward, the methanol-soaked materials were refined from plant debris by filtration and evaporated under vacuum in a rotatory evaporator. Finally, the dried extract was preserved at −20 °C for later use.

### 2.3. Experimental Birds

A total of 105 one-day-old Cobb broiler chickens (Cobb 500) were obtained from Faculty of Agriculture, Mansoura University, Egypt. Chicks were reared in clean floor pens (each one measured 0.5 m^2^ with 0.65 m height; one replicate (3 chicks)/pen). Each pen was covered with wood shaving as litter material at 5 cm depth. All pens had similar feeding and drinking equipment. In the first week, the temperature was set at 32 ± 1 °C and then decreased weakly by 1 °C to reach 25 °C, which was maintained until the end of the study. The relative humidity was kept at 60–70%. All broiler chicks were offered ad libitum access to water and feed. The basal diet was formulated according to Broiler Performance & Nutrition Supplement of Cobb 500 broilers [35]. Diets were adjusted for three programs, involving a starter diet (1–10 days), a growing diet (11–22 days), and a finisher diet (23–42 days). The feed constituents and chemical composition of the basal diet are presented in Table 1. The designed protocol for this experiment was accepted by the Animal Ethics Committee of the Faculty of Veterinary Medicine, Mansoura University, Mansoura, Egypt (Approval No. R/76).

### 2.4. Experimental Design

**Figure 1:**
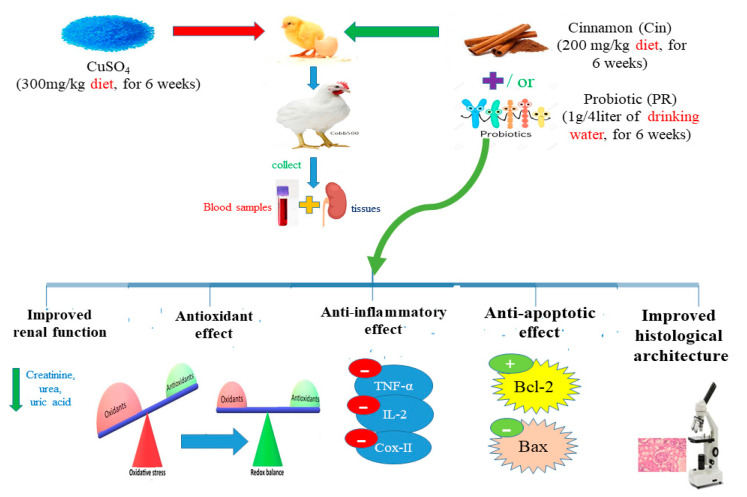
The experimental design and the possible mechanisms of Cin and probiotic preventing Cu-caused renal injury in broilers.

The experimental design is shown in Figure 1. The chicks were randomly allocated into 7 groups (total 15 chicks 15/group) with 5 replicates for each group (3 birds × 5 replicates). The control group was fed a basal diet every day. Cin group was fed a basal diet supplemented with 200 mg cinnamon extract/kg diet daily [34]. PR group was fed a basal diet, and the PR was provided in drinking water daily at a dose of 1 g/4 L of water (following the instructions of the manufacturer in the enclosed pamphlet). Cu group was offered daily a basal diet containing 300 mg/kg CuSO_4_ [9]. Cu + Cin group received a basal diet supplemented with CuSO_4_ and cinnamon extract at the abovementioned dose levels. Cu + PR group was given a basal diet containing CuSO_4_, and the PR was supplied in drinking water daily. Cu + Cin + PR group received a combination of CuSO_4_, Cin, and PR in the same previous manner and doses. The whole experiment lasted 6 weeks, during which the birds were examined daily for signs of illness. The study was divided into two time points at 3 and 6 weeks when sampling.

### 2.5. Sample Collection

Five chickens in each group were selected randomly (*n* = 5) at 3 and 6 weeks. Blood samples were harvested in plane test tubes from the wing veins of these selected chickens. The serum was separated by centrifugation at 3000× *g* and stored at −80 °C for further estimation of kidney function biomarkers and serum immunoglobulins. Later, chickens were euthanized with sodium pentobarbital (30 mg/kg BW). The kidney was collected and rinsed with ice-cold 0.9% NaCl solution. The kidney tissue was divided in to three parts, the first part was homogenized in cold phosphate buffer saline (PBS) (pH 7.4), and centrifuged at 3000× *g*. The supernatant was used for measuring the oxidative stress biomarkers. The second part was kept at −80 °C for performing quantitative real time polymerase chain reaction (real-time PCR) test for gene expression analysis. The third part was preserved in 10% formalin for histopathological and immunohistochemical investigation.

### 2.6. Measurement of Cu Content in the Kidney

Briefly, 0.5 gm kidney tissue sample was macerated and digested with a mixture of 3 mL concentrated nitric acid (65%) and 1.5 mL concentrated perchloric acid (70%). Then, this mixture was incubated for the whole night in a water bath set at 53 °C to achieve complete digestion of the sample. The resultant solution was filtered after cooling at room temperature, and then the filtrate was diluted with 20 mL deionized water. The renal concentration of Cu was measured using flame atomic absorption spectrophotometer (Buck Scientific 210 VGP, Inc., Norwalk, Connecticut, CT, USA) at a wavelength of 324.7 nm, according to the method of AOAC [37].

### 2.7. Biochemical Analysis

#### 2.7.1. Serum Renal Parameters

The serum levels of urea, creatinine, and uric acid were determined spectrophotometrically utilizing assay kits procured from BioMed Co. (Cairo, Egypt, cat. no. URE118200), Human Co. (Wiesbaden, Germany, cat. no. 10051), and Spinreact Co. (Girona, Spain, cat. no. MD41001), respectively.

#### 2.7.2. Estimation of Serum Immunoglobulins

ELISA ready-made kits provided by Roche Diagnostics Co. (Indianapolid, Indiana, IN, USA) were used to measure the serum levels of Immunoglobulin M (IgM, REF; 035071190), and Immunoglobulin G (IgG, REF; 03507432).

#### 2.7.3. Oxidative Stress and Lipid Peroxidation Markers in Renal Tissues

The lipid peroxide (malondialdehyde, MDA) concentration in the homogenized renal tissue was measured using a spectrophotometer based on the technique previously described by Satoh [38]. The activities of enzymatic antioxidant parameters, including catalase (CAT) and superoxide dismutase (SOD), were evaluated spectrophotometrically, as mentioned by Claiborne and Sun et al. [39,40], respectively. Moreover, glutathione (GSH), the nonenzymatic antioxidant index, was assessed according to the directions of Beutler [41].

### 2.8. Gene Expression Analysis of Cytokines and Apoptosis-Related Genes by Real-Time PCR

#### 2.8.1. Total Extraction of RNA and Synthesis of cDNA

RNA was extracted from the collected renal tissues employing the QIAamp RNeasy Mini kit (Qiagen, Germany, GmbH) following the manufacturer’s protocol. The concentration of the isolated RNA was measured utilizing spectrophotometric NanoDrop^®^ (ND-1000). The obtained RNA was reverse transcribed to cDNA, applying the manufacturer’s instructions of QuantiTect Reverse Transcription kit (Qiagen, Heidelberg, Germany).

#### 2.8.2. Quantitative Real-Time PCR

The relative expression of mRNA levels of tumor necrosis factor alpha (TNF-α), interleukin (IL)-2, IL-10, Bax, and Bcl-2 was detected for each sample with a Rotor-Gene Q cycler real-time PCR machine (Qiagen, Heidelberg, Germany) using SYBR Green QuantiTect PCR kits (Qiagen, Germany). The primer designs of the target genes are presented in Table 2. β-Actin was utilized as the internal reference. The conditions of real-time PCR were as follows: first denaturation at 94 °C for 15 min for 40 cycles, then initial heat activation at 94 °C for 15 s; primers annealing at 60 °C for 30 s for Bax and Bcl-2 genes, 59 °C for 1 min for IL-2, 60 °C for 1 min for both IL-10 and TNF-α genes, and 51 °C for 30 s for β actin; and finally, elongation at 72 °C for 30 s. The relative fold changes in the mRNA expression of the investigated genes were estimated as recorded by Yuan et al. [42] through the comparative 2^−ΔΔCt^ method (Ct: cycle threshold).

### 2.9. Renal Histopathological Assessment

Renal tissues were fixed in 10% formalin. Then, standard histological procedures were applied, including dehydration using serial ascending concentrations of ethanol, clearance with xylene, and embedding in paraffin wax. Later, the paraffin blocks were cut at 4 µm thickness, and hematoxylin and eosin (H&E) was used for staining, as described by Bancroft and Layton [48]. The slides were investigated using a light microscope. A semiquantitative scoring of renal lesions was carried out as declared by Gibson-Corley et al. [49] with some modifications. Lesions in 15 fields chosen randomly from each section for each bird were identified, and their mean was calculated. A blinded method was used for lesion scoring [Score scale: 0 = normal; 1 ≤ 25%; 2 = 26–50%; 3 = 51–75%; 4 = 76–100%]. The evaluation of renal lesions depended on the ratio of tubular degeneration and congestion.

Moreover, Masson’s trichrome staining was performed on the kidney sections collected at 6 weeks of the experiment to analyze the presence and extent of fibrosis. The slides were photographed and analyzed under light microscope. Image J software (National Institutes of Health, Bethesda, MD, USA) was used to quantify the percent of the area with fibrosis.

### 2.10. Immunohistochemistry

The immunohistochemical staining of cyclooxygenase-II (COX-II) in the renal sections was conducted following the protocol of Noreldin et al. [50]. In brief, the kidney sections were deparaffinized (in xylene) and rehydrated utilizing sequent ascending dilutions of alcohol. After boiling in 10 mM citrate buffer (pH 6.0) for 0.33 h for antigen unmasking, the sections were preserved at 25 °C for 0.33 h and washed with distilled water. Then, the endogenous peroxidase activity was abolished with 3% H_2_O_2_ in 100% methanol at 4 °C for 0.5 h, before the slides were washed with phosphate buffered saline (PBS). Following this, 10% normal blocking serum was added to the slides for 1 h at room temperature. Thereafter, the slides were incubated overnight at 4 °C with the primary antibody for COX-II (Monoclonal rabbit anti-COX-II at dilution 1:100; ThermoFisher Scientific, Cat: RM-9121-S0, Fremont, 140 CA). Afterward, the slides were subjected to biotinylated goat antirabbit IgG antiserum (Histofine kit, Nichirei Corporation, Tokyo, Japan) for 1hr, and they were rinsed with PBS. Eventually, the streptavidin–peroxidase conjugate (Histofine kit, Nichirei Corporation, Tokyo, Japan) was applied to the slides for 0.5 h. For visualizing the immune reaction, 3, 3′-diaminobenzidine tetrahydrochloride (DAB)–146 H_2_O_2_ solution (pH 7.0) was added for 3 min. The slides were rinsed in distilled water, and hematoxylin was used as a counterstain. A digital camera (Leica EC3, Leica, 148 Germany) connected to a microscope (Leica DM500, Leica, Germany) was used for picking up photomicrographs of the sections. The intensities of immunostaining were quantified using the Image J software (National Institutes of Health, Bethesda, 150 MD, USA). The inverse mean density was assessed as mentioned by Vis et al. [51] in 15 fields selected in a blinded way from various sections of 5 birds in every group.

### 2.11. Growth Parameter Measurements

The growth performance and feed consumption were evaluated by calculating the body weight gain (BWG), feed intake (FI), and feed conversion rate (FCR) of each replicate all over the study period using the following equations, according to Wagner et al. [52]

Weight gain (g) = Mean final weight (g) − Mean initial weight (g)(1)

Feed conversation ration (FCR) = feed consumption (g)/weight gain(g)(2)

### 2.12. Statistical Analysis

Data are exhibited as mean ± SEM. Normality of the data was verified by applying Shapiro Wilk test. The results of growth performance, survival rate, biochemical parameters, gene expression levels, percent of area fibrosis, and immunohistochemical investigation for different experimental groups were compared using one-way analysis of variance (ANOVA), followed by Tukey’s multiple range post hoc test. *p* < 0.05 was considered statistically significant. Data of histopathological scoring was analyzed using Kruskal–Wallis followed by Dunn’s test to compare all means. A *p* < 0.05 indicated statistical significance. Statistical comparison was performed utilizing Statistical Package for Social Science (SPSS), version 20 (SPSS Inc., Chicago, IL, USA) for Windows.

## 3. Results

### 3.1. Cu Concentration in Renal Tissues

**Figure 2:**
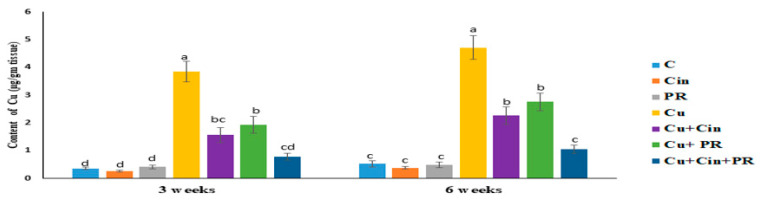
Concentrations of copper in renal tissues of chickens following treatment with cinnamon (200 mg/kg diet), probiotic (1 g/4 L drinking water), and CuSO_4_ (300 mg/kg diet) either individually or concurrently for 3 weeks or 6 weeks. Data are presented as mean ± SEM (*n* = 5 chickens). Each bar carrying different letters is significantly different (*p* < 0.05). C, control; Cin, cinnamon extract; PR, probiotic; Cu, copper.

Figure 2 reveals that Cu content in renal tissues slightly increased with the increase in the time of exposure. Compared to the control group, the Cu concentration in kidney elevated significantly (*p* < 0.05) in chickens that received CuSO_4_. On the other hand, Cu content in Cu + Cin and Cu + PR groups was significantly lower (*p* < 0.05) than the Cu group. Furthermore, no significant difference in the Cu level in renal tissues was observed between Cu + Cin + PR group and the control one.

### 3.2. Serum Renal Injury Biomarkers

The biochemical serum investigations at 3 and 6 weeks elucidated that Cin group and PR group didn’t display significant alterations in all tested parameters, compared to control group. In contrast, the serum levels of creatinine, urea, and uric acid were significantly higher (*p* < 0.05) in CuSO_4_-treated group than the control one at 3 weeks (452%, 168%, and 120%, respectively) and 6 weeks (1078%, 317%, and 215%, respectively). However, treatment with Cin extract, PR, and their combination significantly decreased creatinine, urea, and uric acid serum concentrations in Cu + Cin, Cu + PR, and Cu + Cin + PR groups compared to Cu group (*p* < 0.05) (but still higher than the control group) (Table 3).

### 3.3. Serum Immunoglobulin Levels

A significant decrease (*p* < 0.05) in IgM and IgG serum levels was observed in CuSO_4_ treated chickens at 3 weeks (54% and 68%, respectively) and 6 weeks (68% and 79%, respectively), compared to control chickens. However, Cu + Cin-, Cu + PR-, and Cu + Cin + PR-treated chickens exhibited significant elevation (*p* < 0.05) in serum IgM and IgG in comparison with Cu-treated chickens at any time (Table 4).

### 3.4. Oxidative Stress and Antioxidant Markers in Renal Tissues

**Figure 3:**
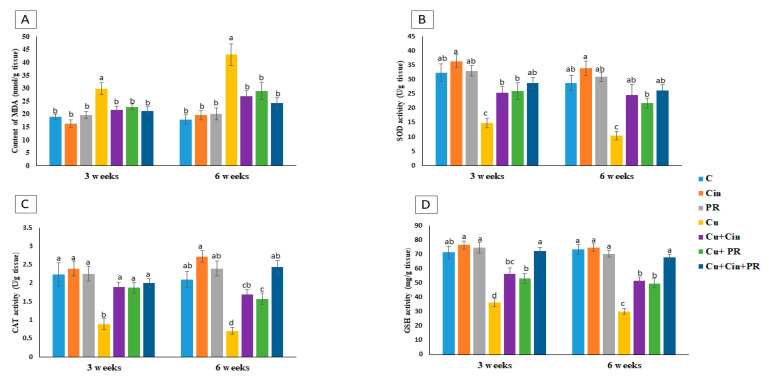
The effect of cinnamon and/or probiotic treatment on renal tissue lipid peroxidation and activities of antioxidant enzymes in copper intoxicated chickens: (**A**) The MDA content, (**B**) the SOD activity, (**C**) the CAT activity, and (**D**) the concentration of GSH. Data are expressed as mean ± SEM (*n* = 5 chickens). Each bar carrying different letters is significantly different (*p* < 0.05). C, control; Cin, cinnamon extract; PR, probiotic; Cu, copper.

As depicted in Figure 3, the level of kidney lipid peroxide (MDA) was significantly elevated (*p* < 0.05) in CuSO_4_-exposed chickens compared with the control group at the two sampling time points (57% and 141% at 3 and 6 weeks, respectively). Contrarily, the renal activities of SOD, CAT, and the concentration of GSH were significantly reduced (*p* < 0.05) in Cu group compared with the control group at 3 weeks (54%, 60%, and 49%, respectively) and 6 weeks (63%, 66%, and 59%, respectively). Meanwhile, the administration of Cin extract or PR contributed to remarkable decline in renal MDA level and increase in SOD, CAT, and GSH activities as indicated in Cu + Cin group and Cu + PR group, relative to Cu group (*p* < 0.05). Moreover, Cu + Cin + PR group didn’t exhibit significant differences in the renal lipid peroxide and antioxidative markers compared to the control group at any time point.

### 3.5. Expression of Cytokines and Apoptosis-Related Genes

**Figure 4:**
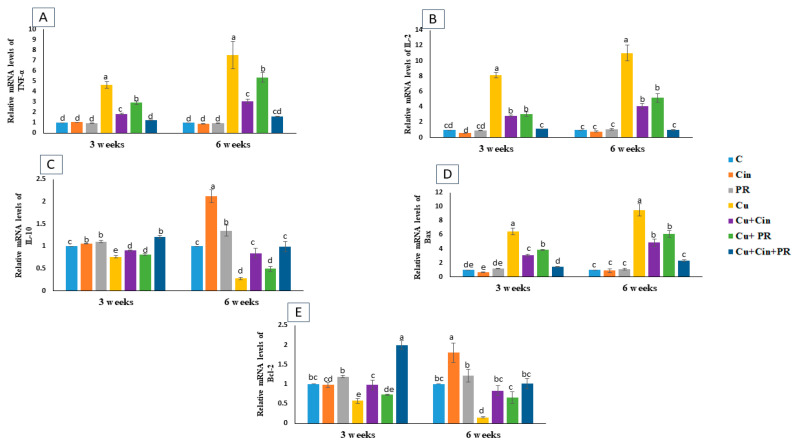
Relative mRNA expression of tumor necrosis factor alpha (TNF-α) (**A**), interleukin (IL)-2(**B**), IL-10 (**C**), Bax (**D**), and Bcl-2 (**E**) in renal tissues of chickens in response to administration of cinnamon (200 mg/kg diet), probiotic (1 g/4 L drinking water), and CuSO_4_ (300 mg/kg diet) either individually or concurrently for 3 weeks or 6 weeks. Data are exhibited as mean ± SEM (*n* = 5 chickens). Each bar carrying different letters is significantly different (*p* < 0.05). C, control; Cin, cinnamon extract; PR, probiotic; Cu, copper.

The quantitative real-time PCR (qRT-PCR) findings displayed a significant upregulation (*p* < 0.05) in the mRNA expression of the proinflammatory cytokines (TNF-α and IL-2) and apoptotic gene (Bax) in the renal tissues of Cu group at 3 and 6 weeks of the study with respect to the control group. Conversely, the expression of the renal anti-inflammatory cytokine (IL-10) and antiapoptotic gene (Bcl-2) was statistically downregulated (*p* < 0.05) in Cu group counterweight to the control group at 3 and 6 weeks. However, a marked reduction (*p* < 0.05) in the renal TNF-α, IL-2 and Bax transcription level and a significant elevation (*p* < 0.05) in IL-10 and Bcl-2 expression was recorded in Cu-intoxicated chickens that were treated with either Cin extract or PR in comparison with Cu group. Moreover, no significant difference in expression of cytokines and apoptosis-related genes was detected between Cu + Cin + PR group and the control group at 6 weeks of the experiment (Figure 4).

### 3.6. Histopathological Alterations

**Figure 5:**
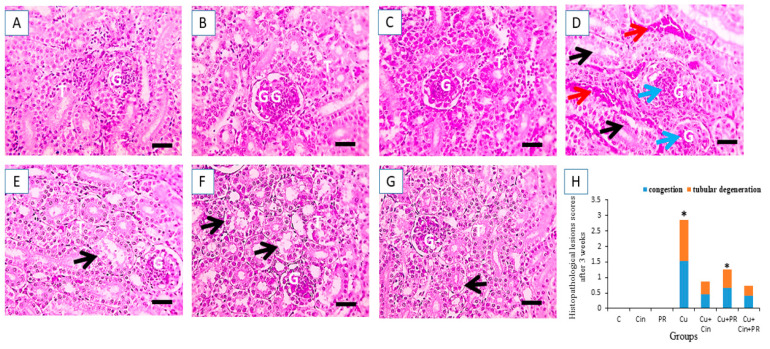
Light photomicrographs of chicken kidney sections (stained with H&E, X 400) after 3 weeks of the experiment. (**A**–**C**) Control group (**C**), cinnamon group (Cin), and probiotic group (PR): showing normal glomeruli (G) and tubules (T). (**D**) Copper group (Cu): revealed degenerated glomeruli (blue arrows) and tubules (black arrows) and congested intertubular capillaries (red arrows). (**E**) Cu + Cin group: showing mildly vacuolated tubular epithelium (black arrows) in few tubules. (**F**) Cu + PR group: showing mildly vacuolated tubular epithelium (black arrows) in few tubules. (**G**) Cu + Cin + PR group: very mildly vacuolated tubular epithelium (black arrows). (**H**) Semiquantitative scoring of renal tubular degeneration and congestion. * Significance compared to control (*p* < 0.05). Scale bar = 50 µm.

The histopathological investigation of the kidney samples collected at 3 weeks of the experiment revealed normal architecture of the glomeruli and tubules, with no histopathological deformities in the control, Cin, and PR groups (Figure 5A–C). However, renal sections from CuSO_4_-exposed chickens showed degenerated glomeruli and tubules with congested intertubular capillaries (Figure 5D). In contrast, the Cu + Cin and Cu + PR groups displayed mildly vacuolated tubular epithelium (Figure 5E,F). Interestingly, the kidney section from Cu + Cin + PR treated chickens exhibited a fairly normal histological structure (Figure 5G).

**Figure 6:**
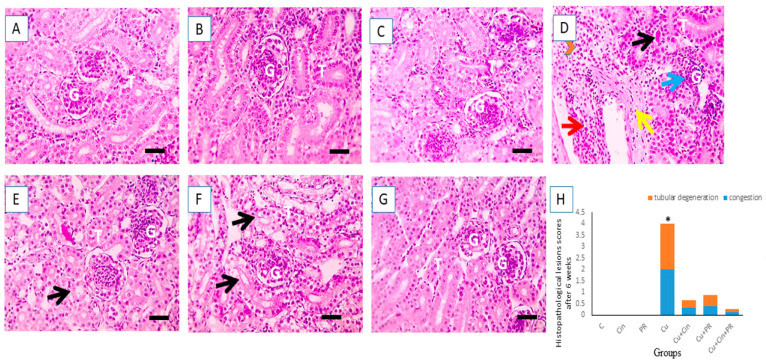
Light photomicrographs of chicken kidney sections (stained with H&E, X 400) after 6 weeks of the experiment. (**A**–**C**) Control group (**C**), cinnamon group (Cin), and probiotic group (PR): showing normal glomeruli (G) and tubules (T). (**D**) Copper group (Cu): elucidating tubular casts (black arrow), atrophied glomeruli (blue arrows), congested blood vessels (red arrows), perivascular hemorrhage (red arrowheads), and fibrosis (yellow arrow). (**E**) Cu + Cin group: showing very mildly vacuolated tubular epithelium (black arrows). (**F**) Cu + PR group: revealing moderately vacuolated tubular epithelium (black arrows). (**G**) Cu + Cin + PR group: Retained normal appearance of tubules and glomeruli. (**H**) Semiquantitative scoring of renal tubular degeneration and congestion. * Significance compared to control (*p* < 0.05). Scale bar = 50 µm.

Figure 5 presented the light photomicrographs of renal tissues obtained from the experimental groups at 6 weeks of the study. Figure 6A–C shows normal renal tissue structure in the control, Cin, and PR groups, while Figure 6D displays tubular casts, atrophied glomeruli, congested blood vessels, perivascular hemorrhage, and fibrosis in the kidneys of Cu-exposed birds. Figure 6E reveals very mildly vacuolated tubular epithelium in renal tissue of Cu + Cin treated group. Meanwhile, in the kidney section of Cu + PR group, moderately vacuolated tubular epithelium was noted (Figure 6F). Furthermore, the histopathological analysis of Cu + Cin + PR group elucidated retained normal appearance of tubules and glomeruli (Figure 6G).

**Figure 7:**
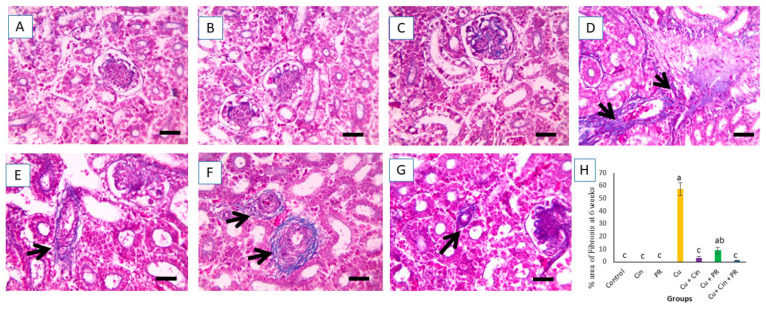
Light photomicrographs of chicken kidney sections (stained with Masson’s trichrome, X 400) after 6 weeks of the experiment. (**A**–**C**) control group (**C**), cinnamon group (Cin), and probiotic group (PR): with no fibrosis. (**D**) Copper group (Cu): elucidating interstitial fibrosis (black arrow). (**E**) Cu + Cin group: showing significant decrease in fibrosis than Cu group (*p* < 0.05). (**F**) Cu + PR group: revealing slight reduction in fibrosis compared to Cu group. (G) Cu + Cin + PR group: presenting significant decrease in fibrosis than Cu group (*p* < 0.05). (**H**) Percent of area with fibrosis. Data are expressed as mean ± SEM. Each bar carrying different letters (a,b,c) is significantly different (*p* < 0.05). Scale bar = 50 µm.

The microscopic pictures of Masson’s trichrome-stained renal sections of different groups at 6 weeks showed no fibrosis in the control, Cin, and PR groups (Figure 7A–C). Renal sections from Cu group showing interstitial fibrosis (Figure 7D). The fibrosis was significantly decreased (*p* < 0.05) in the renal sections of Cu + Cin treated group (Figure 7E), while it was slightly decreased in Cu + PR group (Figure 7F). Furthermore, the renal sections from Cu + Cin + PR-treated chickens revealed marked decrease in the fibrosis compared to Cu group (*p* < 0.05).

### 3.7. Immunohistochemical Findings

**Figure 8:**
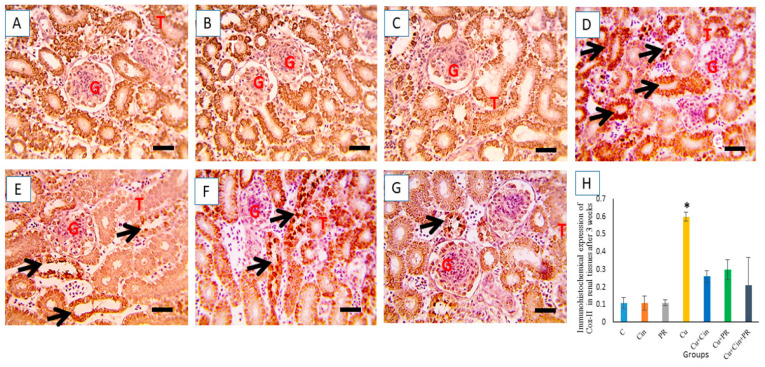
Immunohistochemical staining for COX-II in the chickens’ kidney after 3 weeks of the experiment. (**A**–**C**) control group (**C**), cinnamon group (Cin), and probiotic group (PR): showing minimal positive bright brown tubular expression. (**D**) Copper group (Cu): revealing increased positive bright brown tubular COX-II expression (black arrow). (**E**,**F**) Cu + Cin, and Cu + PR groups: elucidating moderate COX-II reaction. (**G**) Cu + Cin + PR group: exhibiting significant reduction of COX-II-positive cells. (**H**) Representing the quantification of COX-II in the renal tissues in different groups. Data are expressed as mean ± SEM. * Significance compared to control (*p* < 0.05). Scale bar = 50 µm. G, glomeruli; T, tubules.

The microscopic pictures of immunostained renal sections against COX-II after 3 weeks of the experiment showed minimal positive bright brown tubular expression in control, Cin, and PR groups (Figure 8A–C). On the contrary, increased positive bright brown tubular COX-II expression was observed in Cu group (*p* < 0.05) (Figure 8D). Meanwhile, CuSO_4_-intoxicated chickens that received either Cin (Figure 8E) or PR (Figure 8F) showed moderate COX-II reaction. Moreover, significant reduction (*p* < 0.05) of COX-II-positive cells was observed in Cu + Cin + PR group relative to Cu group (Figure 8G).

**Figure 9:**
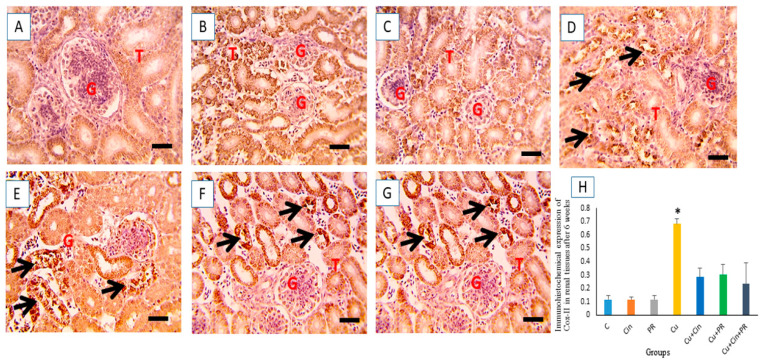
Immunohistochemical staining for COX-II in the chickens’ kidney after 6 weeks of the experiment. (**A**–**C**) control group (**C**), cinnamon group (Cin), and probiotic group (PR): showing minimal positive bright brown tubular expression of COX-II. (**D**) Copper group (Cu): strong positive COX-II immune staining (black arrow). (**E**,**F**) Cu + Cin, and Cu + PR groups: displaying moderate COX-II reaction. (**G**) Cu + Cin + PR group: declaring significant reduction (*p* < 0.05) in COX-II-positive cells compared to Cu group. (**H**) Showing the quantification of COX-II in the renal tissues in various groups. Data are presented as mean ± SEM. * Significance compared to control (*p* < 0.05). Scale bar = 50 µm. G, glomeruli; T, tubules.

After 6 weeks of the study, the immunohistochemical evaluation of the renal tissues presented minimal positive bright brown tubular expression of COX-II in control, Cin, and PR groups (Figure 9A–C). On the other hand, strong positive COX-II immune staining was recorded in Cu group (Figure 9D). However, moderate Cox-II reaction was noticed in Cu + Cin and Cu + PR groups (Figure 9E,F). The COX-II expression was significantly lower (*p* < 0.05) in combination group (Cu + Cin + PR) than in CuSO_4_-intoxicated chickens treated with Cin extract or PR, alone (Figure 9G).

### 3.8. Survival Rate and Growth Performance

The survival rate of the birds was significantly lower (*p* < 0.05) in CuSO_4_-treated chickens in comparison with all other experimental groups. In addition, Cu group demonstrated the lowest growth performance (*p* < 0.05). Meanwhile, Cu + PR groups exhibited significant elevation (*p* < 0.05) in final body weight (FBW) and BWG than the Cu group. On the contrary, FCR was significantly lower in Cu + Cin and Cu + PR groups comparing to CuSO_4_ treated group. Moreover, the concurrent administration of Cin extract and PR to the CuSO_4_-intoxicated chickens showed significant higher growth performance (*p* < 0.05) than when each one was administered with CuSO_4_, alone (Table 5).

## 4. Discussion

The kidney is considered more vulnerable to copper poisoning because of its filtration and excretion role [9,53,54]. Accumulation of excessive amounts of Cu inside the cells may perturb the redox homeostasis and eventually lead to a sequence of deleterious effects, for instance, inflammation, degeneration, apoptosis, and necrosis [55]. The current investigation assessed the nephrotoxicity of copper poisoning and the ameliorative effect of Cin extract and/or PR administration against Cu intoxication in broiler chickens. The present research declared that dietary exposure of chickens to CuSO_4_ at 300 mg/kg induced marked renal injury, as indicated by the elevation of serum concentrations of renal function parameters (creatinine, urea, and uric acid) in a time-dependent manner at 3 and 6 weeks of the experiment. Creatinine and urea are accounted as reliable indicators for diagnosis of kidney impairment, as they are nitrogenous end products of catabolic process that are generally eliminated by the kidney. Our results lie in the same line with those of Dai et al. [54], who reported that the administration of CuSO_4_ to mice at 200 mg/kg for 28 days was associated with increase in the serum levels of creatinine and urea.

Moreover, remarkable pathological changes were recorded in renal tissues of chickens exposed to CuSO_4_, including tubular casts, atrophied glomeruli, congested blood vessels, perivascular hemorrhage, and fibrosis. These structural alterations supported the noticed changes in serum renal function biomarkers and were parallel to those announced by Dai et al. [54], who observed tubular degeneration, cast formation, and glomerular degeneration in mice which received CuSO_4_. Similarly, Wang et al. [9] demonstrated that exposure of chickens to CuSO_4_ at a dose of 300 mg/kg diet for 12 weeks resulted in alterations in renal histoarchitecture observed as degeneration and necrosis of tubular cells. Atrophied glomeruli and tubular casts suggest impaired glomerular filtration and explain the deteriorations in kidney performance.

These renal injuries probably attributed to oxidative stress, which disturbs cell membrane structure and performance, causing tissue damage. Several research groups have proved that heavy metals can upset the oxidant–antioxidant balance with consequent generation and aggregation of free radicals and, eventually, oxidative stress [6,10]. Oxidative stress is a state that arises from the disequilibrium between the formation of free radicals and antioxidant defenses [56]. SOD and CAT are considered the first line of protection in the antioxidant system, which serves as a scavenger for free radicals [57]. SOD, a main enzyme to eradicate oxyradicals, acts as a catalyst for the transformation of superoxide radicals to hydrogen peroxide. CAT, an enzyme, exists in peroxisomes and aids in the elimination of hydrogen peroxide [58], while, GSH is a nonenzymatic molecule, which can quench a broad diversity of reactive species [59]. Thus, SOD, CAT, and GSH can be regarded as reliable markers for estimating the antioxidant capacity. Further, MDA is a valuable indicator for oxidative damage and free radicals, since it is the outcome of lipid peroxidation [60]. In this study, the remarkable elevation of MDA content in renal tissues at 3 and 6 weeks of Cu exposure indicates an augmentation in lipid peroxidation, while the statistical reduction in the activities of SOD and CAT and the concentration of GSH in the kidneys of Cu-poisoned chickens relative to the control group points out their exhaustion in removing ROS. These results indicated that excess Cu could reduce the antioxidant capacity and cause oxidative stress. Our findings were in accordance with those of previous reports [6,9,54,61].

Further, the immunohistochemical analysis demonstrated overexpression of COX-II in renal tissues of CuSO_4_-intoxicated chickens. COX-II, an enzyme, plays a pivotal role in the synthesis of prostaglandins that mediate the inflammatory reactions [62]. The exaggerated COX-II expression in Cu group may be correlated to the inflammation caused by CuSO_4_ overdose. Former investigation revealed that inflammation is one of the potential mechanisms underlying copper-induced nephrotoxicity [9]. Inflammation is considered a major consequence of oxidant–antioxidant imbalance [63]. It has been determined that overproduction of ROS enhances the generation of nuclear factor–kappa (NF–kB), in addition to other cytological signaling events, which consequently increase the expression of proinflammatory genes such as COX-II, IL-1β, IL-6, and TNF-α [64,65]. In this regard, our research elucidated a sustained increase in the mRNA levels of proinflammatory cytokines, including TNF-α and IL-2, in the kidney after Cu exposure. Lipid peroxidation, which leads to reduced glomerular filtration rate, may account for the excessive cytokines recruitment. On the contrary, the results exhibited a significant decline in the expression of the renal anti-inflammatory cytokine (IL-10). These findings coincide with a preceding study [9].

Additionally, the present study revealed a significant increase in the transcription of Bax mRNA and a decrease in the transcription of Bcl-2 mRNA in the renal tissues of Cu- poisoned chickens. Bax, proapoptotic protein, and Bcl-2, antiapoptotic protein, are members of Bcl-2 family which modulates the mitochondrial-dependent apoptotic pathways within the cells [66]. Apoptosis is a physiological process of cell self-killing that is accountable for normal growth and homeostasis in multicellular organisms during their whole lifespan [67]. It has been documented that the excessive release of free radicals and oxidative stress can trigger cell apoptosis [68]. Previous literatures indicated that excess ROS and Bax molecule can impair the mitochondrial membrane permeability, leading to the expulsion of cytochrome c (Cyt c) in to the cytosol, which, in turn, attaches to apoptotic activating factor 1 (APAF-1), resulting in caspase stimulation and, ultimately, apoptosis [69,70,71]. On the other hand, Bcl-2 is an antiapoptotic protein that suppresses the efflux of Cyt c from mitochondria into cytoplasm via antagonizing the apoptotic molecules and maintaining the mitochondrial membrane integrity [72]. In support of our findings, Dai et al. [54] observed a significant upregulation in the mRNA expression of Bax and caspase-3 in the kidneys of mice exposed to CuSO_4_ and thus concluded that the mitochondrial apoptotic pathway was implicated in the nephrotoxicity caused by CuSO_4_. Similarly, Kawakami et al. [69] mentioned that excessive exposure to Cu can cause apoptosis through augmenting the expression of Bax, Bad, Cyt c, caspase-3, and caspase-9 in PC12 cells. Moreover, recent reports declared that CuSO_4_ caused apoptosis in the liver cells of chickens and rats [73,74,75].

Interestingly, the results of the current research demonstrated the protective effect of Cin extract against Cu-induced nephrotoxicity. This ameliorative role of Cin extract was reflected from the restoration of normal control concentrations of serum renal function parameters; immunoglobulin levels; renal tissue antioxidant markers; histological structure; mRNA levels of TNF-α, IL-2, IL-10, Bax, and Bcl-2; and the expression of COX-II enzyme in chickens received Cin extract at 200 mg/kg diet. These results were parallel to the findings of many authors who proved the role of Cin in preventing the nephrotoxicity, caused by bisphenol, octylphenol, cypermethrin, acetaminophen, oxytetracycline, and diclofenac sodium [17,27,76]. Morgan et al. [17] have revealed that the protective action of Cin extract against renal oxidative injury may be attributed to its enhancing effect on the antioxidant enzymes and inhibitory action on ROS synthesis. Similarly, previous researchers have recorded the antioxidant action of Cin in vitro and in vivo [77,78]. The antioxidant activity of Cin may be owed to its phenolic and flavonoids components, which serve as free radicals scavengers, redox active transition metal chelators, and enzyme modulators [18]. Moreover, former investigators have announced that Cin exerted an anti-inflammatory action in different organs via inhibiting the expression of inducible nitric oxide synthase (iNOS) and COX-II [27,79]. The histopathological and immunohistochemical findings emphasized the guarding effect of Cin extract against renal damage caused by high dose of CuSO_4_. In addition, this report elucidated that the administration of Cin extract to Cu-poisoned chickens exhibited remarkable increase in IgM and IgG serum levels compared to chickens treated with Cu, only. These findings were consistent with Niphade et al. [80], who reported that Cin extract could trigger the humoral immunity in Swiss albino mice.

The widespread usage of PR as natural alternative medicines in pharmaceutical products and feed additives encourages the researchers to investigate the capability of these living, nonharmful organisms to prevent germs and poisons adhesion to surfaces. Hence, our research studied the potential protective effect of PR against renal damage caused by Cu overdose in chickens. The administration of PR ameliorated the renal tissue injury, oxidative stress, the elevated mRNA expression of proinflammatory cytokines (TNF-α and IL-2), and apoptotic gene (Bax) and reduced mRNA level of anti-inflammatory cytokines (IL-10) and antiapoptotic gene (Bcl-2), and overexpression of COX-II enzyme in chickens intoxicated by Cu. In concurrence with these results, several studies have recorded the mitigating effect of PR against kidney impairment caused by cadmium and cisplatin [33,81]. It has been declared that PR bacteria could alleviate renal injury by reducing oxidative stress [81]. Prior reports have elucidated that lactobacilli bacteria act as an antioxidant via promoting endogenous antioxidant, modulating the lipid metabolism, and suppressing lipid peroxidation [82,83]. What’s more, some lactobacillus strains have been recognized to possess a complete GSH system which enables them to perform a good guarding action against oxidative stress [81,84,85]. In addition, Zoghi et al. [86] announced that PR lactic acid bacteria have the ability to antagonize toxins by surface binding owed to prominent adhesive features of S-layer-protein in their cell membrane. Previous studies have demonstrated that some lactobacilli can bind and eliminate heavy metals such as lead, cadmium, and copper in vitro [31,32]. Furthermore, it has been proven that PR inhibited the expression of proinflammatory cytokines caused by pathogen invasion in the intestine of mice [87].

In this study, the survival rate and growth performance of broilers in Cu group significantly reduced in comparison to other groups. Similarly, former investigations have reported that treatment of chickens with high concentrations of CuSO_4_ led to remarkable decrease in final body weight, feed intake, and growth rate of broiler chickens [88]. Mehring et al. [89] found that the administration of CuSO_4_ in excess to broiler chickens caused significant increase in the mortality. Nevertheless, the survival rate of broiler chickens in Cu + Cin, Cu + PR, and Cu + Cin + PR groups significantly increased compared to Cu group. The findings are in agreement with Yang et al. [90], who observed that cinnamon oil reduced the mortality caused by coccidiosis in chickens. Additionally, birds in Cu + PR group exhibited a significant enhancement in growth parameters relative to Cu group. The improvement of growth performance by PR supplementation may be related to the activation of intestinal microflora, which suppresses the growth of pathogenic microorganisms, improves intestinal health, and enhances digestibility [91].

Another interesting finding of the present work was that the Cin extract had more remarkable ameliorative action compared to PR against Cu nephrotoxicity. Moreover, this study is, to the best of authors’ knowledge, the first to reveal that concurrent administration of Cin and PR resulted in more pronounced renal protection than when each one is given individually.

In conclusion, Cin extract and PR afforded renal protection against CuSO_4_-induced nephrotoxicity via modulating oxidative stress, inflammation, and cell apoptosis in broiler chickens. Further research is warranted to elucidate the characterization of all Cin active components and to investigate the effects of each constituent exclusively. In addition, future studies are needed to evaluate additional markers in the inflammatory and apoptotic signaling pathways to verify other mechanisms that may be implicated in the protective effects of both Cin and PR against Cu-induced nephrotoxicity.

## Figures and Tables

**Table 1:** Ingredients and chemical composition of the basal diet (as DM).

Ingredients, %	Starter (1 to 10 Days)	Grower (11 to 22 Days)	Finisher (23 to 42 Days)
Yellow corn grain	58.00	62.80	63.60
Soybean meal, 47.5%	34.46	27.40	25.00
Corn gluten, 60%	1.5	21.80	3.00
Wheat bran	--	1.00	1.40
Soybean oil	1.75	2.00	3.30
Calcium carbonate	1.10	1.00	1.00
Dicalciumphosphate	1.90	1.70	1.50
Common salt	0.30	0.30	0.30
Premix *	0.30	0.30	0.30
DL–Methionine, 98%	0.18	0.19	0.11
Lysine, Hcl, 78%	0.16	0.16	0.14
Antimycotoxin **	0.10	0.10	0.10
Sodium bicarbonate	0.25	0.25	0.25
**Analyzed Chemical Composition**			
ME ***, Kcal/Kg	3020.56	3090.13	3180.70
CP, %	22.08	20.01	19.04
EE, %	4.25	4.63	5.92
CF, %	1.64	2.62	2.60
Ca, %	1.03	0.93	0.87
Available P, %	0.48	0.44	0.40
Lysine, %	1.35	1.16	1.10
Methionin, %	0.53	0.53	0.45

* Supplied, kg/diet: Vitamin A, 12,000 IU; Vitamin D3, 2200 IU; Vitamin E, 26 IU; Vitamin K3, 6.25 mg; Vitamin B1, 3.75 mg; Vitamin B2, 6.6 mg; Vitamin B6, 1.5 g; Pantothenic acid, 18.8 mg; Vitamin B12, 0.31 mg; Niacin, 30 mg; Folic acid, 1.25 mg; Biotin, 0.6 mg; Fe, 50 mg; Mn, 60 mg; Cu, 6 mg; I, 1 mg; Co, 1 mg; Se, 0.20 mg; Zn, 50 mg; Choline chloride, 500 mg. ME, metabolic energy; CP: crude protein; EE, ether extract; CF, crude fiber; Ca, calcium; P, phosphorus. ** Antimycotoxin, Toxifin dry: Multimycotoxin binder that includes bentonite (1m558) from Kemin Industries, Inc. *** ME, metabolic energy was calculated according to NRC [36].

**Table 2:** Primer sequences of gene analyzed in real-time PCR.

Target Gene	Forward Primer (5′–3′)	Reverse Primer (5′–3′)	References
TNF-α	CCCCTACCCTGTCCCACAA	ACTGCGGAGGGTTCATTCC	[43]
IL-2	TTGGAAAATATCAAGAACAAGATTCATC	TCCCAGGTAACACTGCAGAGTTT	[44]
IL-10	CATGCTGCTGGGCCTGAA	CGTCTCCTTGATCTGCTTGATG	[45]
Bax	TCCATTCAGGTTCTCTTGACC	GCCAAACATCCAAACACAGA	[46]
Bcl-2	ATCGTCGCCTTCTTCGAGTT	ATCCCATCCTCCGTTGTTCT	[46]
*ß*. actin	CCACCGCAAATGCTTCTAAAC	AAGACTGCTGCTGACACCTTC	[47]

**Table 3:** Serum biochemical markers of renal functions in control and experimental groups at 3 and 6 weeks.

Experimental Groups	3 Weeks			6 Weeks		
	Creatinine (mg/dL)	Urea (mg/dL)	Uric Acid (mg/dL)	Creatinine (mg/dL)	Urea (mg/dL)	Uric Acid (mg/dL)
C	0.25 ± 0.03 ^c^	2.21 ± 0.29 ^b^	4.68 ± 0.61 ^c^	0.28 ± 0.04 ^d^	2.26 ± 0.37 ^d^	5.10 ± 0.49 ^cd^
Cin	0.20 ± 0.02 ^c^	1.83 ± 0.34 ^b^	4.16 ± 0.32 ^c^	0.23 ± 0.03 ^d^	2.06 ± 0.25 ^d^	4.32 ± 0.26 ^d^
PR	0.23 ± 0.02 ^c^	2.15 ± 0.19 ^b^	4.84 ± 0.42 ^bc^	0.25 ± 0.04 ^d^	2.62 ± 0.31 ^cd^	4.76 ± 0.37 ^cd^
Cu	1.38 ± 0.21 ^a^	5.94 ± 0.85 ^a^	10.32 ± 0.69 ^a^	3.3 ± 0.37 ^a^	9.44 ± 0.72 ^a^	16.08 ± 1.47 ^a^
Cu + Cin	0.59 ± 0.07 ^bc^	3.48 ± 0.64 ^b^	6.1 ± 0.78 ^bc^	1.42 ± 0.19 ^bc^	4.56 ± 0.44 ^bc^	8.40 ± 0.73 ^bc^
Cu + PR	0.67 ± 0.08 ^b^	3.74 ± 0.33 ^b^	7.48 ± 0.75 ^b^	1.92 ± 0.27 ^b^	6.76 ± 0.69 ^b^	11.06 ± 1.28 ^b^
Cu + Cin + PR	0.36 ± 0.05 ^bc^	2.96 ± 0.42 ^b^	5.04 ± 0.60 ^bc^	0.84 ± 0.14 ^cd^	3.44 ± 0.54 ^cd^	6.82 ± 0.67 ^cd^

Data are expressed as the mean ± SEM (*n* = 5 chickens). ^a,b,c,d^ Different superscripts within each row indicate significant differences (*p* < 0.05). C, control, Cin; cinnamon extract, PR, probiotic; Cu; copper.

**Table 4:** Serum levels of IgM and IgG in control and experimental groups at 3 and 6 weeks.

Experimental Groups	3 Weeks		6 Weeks	
	IgM (mg/dL)	IgG (mg/dL)	IgM (mg/dL)	IgG (mg/dL)
C	11.74 ± 0.93 ^ab^	1.38 ± 0.22 ^ab^	13.62 ± 1.43 ^a^	1.78 ± 0.16 ^ab^
Cin	14.02 ± 1.75 ^a^	1.60 ± 0.18 ^ab^	15.24 ± 1.82 ^a^	2.40 ± 0.27 ^a^
PR	12.56 ±0.89 ^ab^	2.00 ± 0.26 ^a^	13.88 ± 1.16 ^a^	1.88 ± 0.25 ^b^
Cu	5.36 ± 0.68 ^c^	0.44 ± 0.10 ^c^	4.30 ± 0.52 ^b^	0.37 ± 0.05 ^d^
Cu + Cin	10.34 ± 0.76 ^ab^	0.88 ± 0.09 ^bc^	10.84 ± 1.50 ^a^	1.26 ± 0.15 ^bc^
Cu + PR	8.74 ± 0.84 ^bc^	1.06 ± 0.17 ^bc^	9.62 ± 0.65 ^a^	0.89 ± 0.13 ^cd^
Cu + Cin + PR	10.56 ± 1.00 ^ab^	1.24 ± 0.16 ^ab^	12.06 ± 0.94 ^a^	1.39 ± 0.19 ^b^

Data are expressed as the mean ± SEM (*n* = 5 chickens). ^a,b,c,d^ Different superscripts within each row indicate significant differences (*p* < 0.05). C, control, Cin; cinnamon extract, PR, probiotic; Cu; copper.

**Table 5:** Growth performance of broiler chickens treated with copper, cinnamon extract, and probiotic.

Parameters	IW(g/Bird)	FBW (g/Bird)	BWG (g/Bird)	FI (g/Bird)	FCR	Survival %
**C**	42.38 ± 0.17	2608 ± 30.23 ^a^	2565.62 ± 30.27 ^a^	4234 ± 28.39 ^a^	1.65 ± 0.02 ^cd^	100 ± 0.00 ^a^
**Cin**	42.38 ± 0.11	2602 ± 50.06 ^a^	2559.62 ± 49.99^a^	4157 ± 62.96 ^a^	1.63 ± 0.02 ^d^	100 ± 0.00 ^a^
**PR**	42.42 ± 0.10	2718 ± 66.17 ^a^	2676.18 ± 66.10 ^a^	4203 ±55.54 ^a^	1.57 ± 0.02 ^d^	100 ±0.00 ^a^
**Cu**	42.34 ± 0.12	2046 ± 36.96 ^d^	2003.66 ± 36.92 ^d^	3756 ± 31.40 ^c^	1.88 ± 0.03 ^a^	66.60 ± 0.00 ^b^
**Cu + Cin**	42.30 ± 0.11	2167 ± 26.00 ^cd^	2124.70 ± 26.01 ^cd^	3712 ± 27.17^c^	1.75 ± 0.02 ^b^	86.64 ± 8.18 ^a^
**Cu + PR**	42.34 ± 0.12	2191 ± 47.68 ^c^	2148.66 ± 47.75 ^c^	3811 ± 59.13 ^c^	1.78 ± 0.04 ^b^	79.96 ± 8.18 ^a^
**Cu + Cin + PR**	42.26 ± 0.14	2359 ± 41.18 ^b^	2316.74 ± 41.22 ^b^	3987 ± 95.27 ^b^	1.72 ± 0.04 ^bc^	93.32 ± 6.68 ^a^

Values are mean ± SEM (*n* = 5 replicates). ^a,b,c,d^ Different superscripts within each row indicate significant differences (*p* < 0.05). C, control, Cin; cinnamon extract, PR, probiotic; Cu; copper; IW, initial weight; FBW, final weight; BWG, body weight gain; FCR, feed conversion ratio.

## Data Availability

The datasets generated during and/or analysed during the current study are available from the corresponding author on reasonable request.
